# Rescue of an enterotropic Newcastle disease virus strain ZM10 from cloned cDNA and stable expressing an inserted foreign gene

**DOI:** 10.1186/s12896-022-00763-5

**Published:** 2022-12-06

**Authors:** Lei He, Hairong Wang, Zuhua Yu, Chengshui Liao, Ke Ding, Cai Zhang, Chuan Yu, Chunjie Zhang

**Affiliations:** 1 College of Animal Science and Technology/Luoyang Key Laboratory of Live Carrier Biomaterial and Animal Disease Prevention and Control, Henan University of Science and Technology, Luoyang, 471023 Henan China; 2 Animal Diseases and Public Health Engineering Research Center of Henan Province, Luoyang Polytechnic, Luoyang, 471900 Henan China

**Keywords:** Newcastle disease virus, Reverse genetics system, Enterotropic, Red fluorescent protein

## Abstract

**Background:**

Newcastle disease virus (NDV) strain ZM10, a typical enterotropic avirulent vaccine strain, has been widely used in China for chickens against Newcastle disease. To elucidate its enterotropic mechanism and develop recombiant multivalent vaccines based on it, the reverse genetics system for NDV ZM10 is an indispensable platform.

**Results:**

A full-length cDNA clone of NDV ZM10 and three supporting plasmids were constructed using the ligation-independent cloning method. Recombinant NDV rZM10 was successfully rescued after these plasmids were co-transfected into BHK-21 cells. Besides, the recombinant virus rZM10-RFP encoding the red fluorescent protein was generated by inserting the RFP gene into the full-length clone of NDV between the P and M genes. These rescued viruses were genetically and biologically identical to the parental strain and showed similar growth kinetics.

**Conclusion:**

The recovery system of NDV ZM10 strain was established, and can be used as a foundation for research on the enterotropic mechanism and development of multivalent vaccines against viral diseases of livestock and poultry.

**Supplementary Information:**

The online version contains supplementary material available at 10.1186/s12896-022-00763-5.

## Background

Newcastle disease (ND) is a highly contagious avian disease affecting all species of birds that can cause tremendous socio-economic impact on the poultry industry [1]. The etiological agent of ND, Newcastle disease virus (NDV), belongs to genus *orthoavulavirus* of the *Paramyxoviridae* family. Strains of NDV are categorized into four pathotypes depending on the pathological severity in chickens: highly virulent (velogenic), intermediate (mesogenic), low-virulent (lentogenic), and asymptomatic enteric [2, 3]. Velogenic viruses are further subdivided into viscerotropic and neurotropic based on the severe symptom types of intestinal lesions and neurologic signs, respectively [4]. The infection of chicken with velogenic NDV strains causes a severe acute disease with high morbidity and mortality, characterized by the organs impairment of neurological, gastrointestinal, reproductive, while lentogenic and asymptomatic enteric NDV strains often cause subclinical infection with no symptom or mild respiratory signs and have been used as live attenuated vaccines worldwide [5].

NDV is a non-segmented, negative sense single stranded RNA virus and comprises one of the three genome size:15,186, 15,192 and 15,198 containing six genes and encoding at least six proteins, the nucleocapsid protein(NP), phosphoprotein (P), matrix protein (M), fusion protein(F), hemagglutinin-neuraminidase protein (HN), and largepolymerase protein (L) arranged in the order of 3′leader-NP-P-M-F-HN-L-5′trailer [6, 7]. The ZM10 strain was isolated from the intestine of broiler chickens showing no signs of respiratory disease, and replicates both in the respiratory and intestinal tract, with preference for the intestine. The ZM10 virus is an avirulent strain which has no detectable respiratory reaction in chickens regardless of the administration route [8, 9]. It is widely used in China as a vaccine strain and could induced solid immunity in inoculated chickens with high titer NDV antibody in the serum and robust mucosal response in addition to a systemic response [8].

Since the first reverse genetics system for NDV has been established in 1999 [10], many NDV vaccine strains such as Anhinga LaSota [10, 11]; Beaudette C [12]; Italien [13]; Mukteswar [14]; Banjarmasin/010/10 [15]; VG/GA [16]; TS09-C [17]; NA-1 [18] and R2B [19] have been successfully rescued. The advent of the vectors of NDV strains not only allows us to study the pathogenesis of NDV but also provides a powerful tool to develop new vaccines to express foreign genes against the diseases of poultry and other animals. It is necessary to develop the reverse genetics system for the ZM10 strain as it has been proved to be an excellent NDV vaccine candidate. Therefore, the present work describes the development of a reverse genetics system based on the full-length ZM10 cDNA. Furthermore, the recombinant virus rZM10-RFP, which expresses a reporter red fluorescent protein RFP, is especially useful for studying its enterotropic mechanism and developing it as a vaccine vector for the delivery of heterologous proteins.

## Methods

### Viruses, cells culture and plasmids

The NDV ZM10 strain was purchased from QYH Biotech Co., Ltd and propagated in 9-day-old SPF chicken embryos. Baby hamster kidney BHK-21 (CCL-10; ATCC) cells and DF-1 cells (CRL-12203; ATCC) were maintained at 37 °C and 5% CO_2_ in Dulbecco’s Modified Eagle Medium (DMEM, ThermoFisher Scientific) supplemented with 10% heat-inactivated fetal calf serum (FBS, Beyotime, China), and antibiotics (100 μg/mL streptomycin and 100 U/mL penicillin). The DF-1 cells were cultured in DMEM containing 10% allantoic fluid (AF) from 10-day-old specific-pathogen-free (SPF) chicken embryos for all subsequent infection experiments unless otherwise indicated. The culture medium was replaced every 3 days. The modified pBluescript plasmid (a gift of Dr Qingzhong Yu) which possess T7 promoter at upstream and HDV Rz sequence at downstream of the multi-cloning sites was used during the vector construction [12].

### Construction of full-length NDV cDNA

To obtain the cDNA, the original ZM10 strain was purified by the limiting dilution method through 9-day-old SPF chicken embryos for 3 passages. During each purification, the HA positive allantoic fluid of the highest dilution was collected and used for the subsequent passage. Viral genomic RNA of the purified virus was isolated with a MiniBEST Viral RNA/DNA Extraction Kit (TaKaRa Bio Inc, Japan). The full length genomic sequence of ZM10 was determined by RT-PCR in which the whole genome were divided into five fragments and the 3′ leader and 5′ trailer sequences of strain ZM10 were determined using 3′-rapid amplification of cDNA ends (3′-RACE) and 5′-RACE, respectively (Data not shown), and the complete genome was submitted in the Genbank database (accession no. OL676769).

**Fig. 1:**
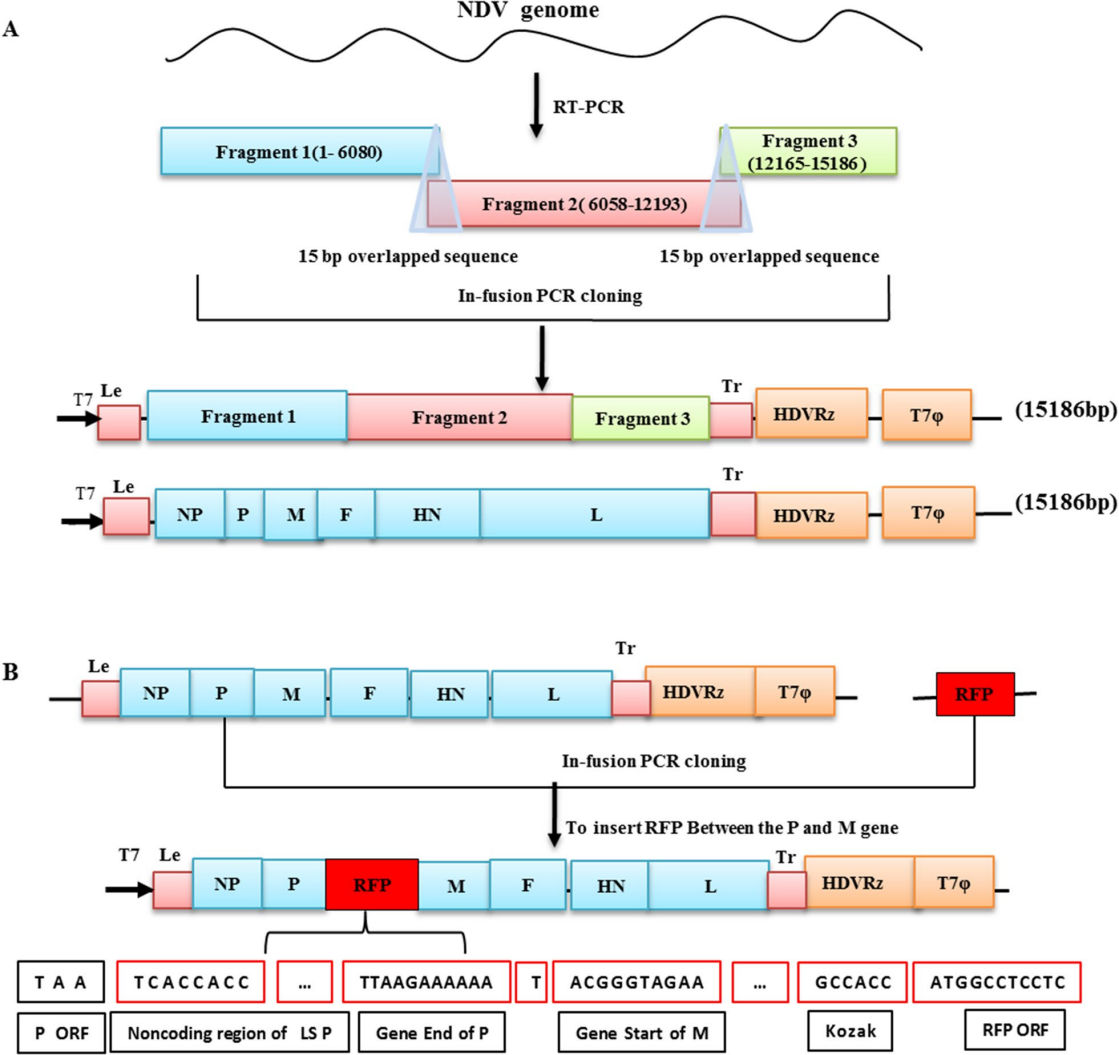
Schematic representation of the construction of full-length cDNA clone of NDV ZM10 strain and rZM10 expressing the RFP gene. **A** The full-length cDNA plasmid of the NDV genome was assembled from three fragments by ligation-independent cloning method. All the three fragments share 15 nt homologous sequence and are shown by different colors: blue (Fragment 1), Red (Fragment 2) and Green (Fragment 3). The full-length clone is under the control of T7 promoter; the fragment Tr was followed by the hepatitis delta virus (HDV) ribozyme and the T7 terminator. **B** Cloning strategy to incorporate the RFP gene into the full length NDV clone of strain ZM10. The RFP cassette was amplified from pLS-RFP vector and sub-cloned into the NDV full-length genome between the P and M gene incorporating the NDV gene-start and gene-end signals as an independent transcription unit (ITU). Kozak. Kozak sequence, ORF: open reading frame

Based on verified sequences of ZM10, the NDV full-length cDNA clone was constructed by three steps of the ligation-independent cloning (LIC) as illustrated in Fig. 1, by using an In-Fusion PCR Cloning Kit (Clontech, Mountain View, CA). Primer sets were designed to contain a 15-nucleotide (nt) overlapping region of homology at their 5′ end to amplify the three cDNA fragments and the linearized vector backbone (Table 1). The viral genome was divided into three segments (Fragment 1, Fragment 2 and Fragment 3) to construct the NDV full-length cDNA clone. All the three fragments were obtained by using the RT-PCR method. For the RT step, briefly, viral genomic RNA of purified ZM10 virus was isolated with a MiniBEST Viral RNA/DNA Extraction Kit (TaKaRa Bio Inc, Japan). cDNAs of Fragment 1, Fragment 2 and Fragment 3 were obtained by using a PrimeScript™ II 1st Strand cDNA Synthesis Kit (TaKaRa Bio Inc, Japan) with the specific primer F1 F, F2 F and F3 F respectively. For the PCR stage, all the three fragments are achieved by using the PrimeSTAR® GXL DNA Polymerase (TaKaRa Bio Inc, Japan) to avoid mutations, as shown in Fig. 1, it Fragment 1 was generated by using PCR with the cDNA of Fragment 1 as template and with primers F1 F and F1 R. Then the Fragment 1 was cloned into the corresponding modified pBluescript vector which also linearized by PCR with specific primers F1 Vet F and F1 Vet R (Table 1), resulting in production of a pZM10-F1 subclone. Subsequently, Fragment 2 was amplified with the primers F2 F and F2 R and cloned into the pZM10-F1 vector linearized by PCR with specific primers and F2 Vec F and F1 R), to obtain the subclone pZM10-F1-F2. Finally, The plasmid pZM10 containing the whole genome was constructed by insert the Fragment 3 into the subclone pZM10-F1-F2 using the above method with the primers F3 F and F3 R for Fragment 3 and F3 Vec F and F2 R for linearized vector pZM10-F1-F2. The plasmid were transformed into Stbl2 cells (Weidi Bio, China) and amplified at 30 °C for 24 h and then purified by using a QIAprep Spin Miniprep kit (Qiagen).

**Table 1:** Primer sequences of the construction of ZM10 full-length cDNA clone

Primer	Primer sequence	Primer name
1^a^	**ACCAAACAGAGAATC**CGTAAG	F1 F
2^a^	**CAAGAGATATGACAG**TTAAAACG	F1 R
3^a^	**CTGTCATATCTCTTG**TATGTGGTATACTTAGCCTGG	F2 F
4^a^	**ATCCAGTCTATGTTG**GAGATTCCCAGCTG	F2 R
5^a^	**CAACATAGACTGGAT**GACGGCATAACTCAGATGAC	F3 F
6^a^	**ACCAAACAAAGATTT**GGTGAATGACAAGAC	F3 R
7^b^	**ACAACCCTGTCCTGC**TTCCTCTG	RFP-F
8^b^	**CGCTCGGGGTGTTGG**ACCTTGGG	RFP-R
9^b^	**CTGTCATATCTCTTG**GGCCGGCATGGTCCCAGCCTCC	F1 Vec F
10^c^	**GATTCTCTGTTTGGT**CCCTATAGTGAGTCGTATTAGCG	F1 Vec R
11 ^c^	**CAACATAGACTGGAT**GGCCGGCATGGTCCCAGCCTCC	F2 Vec F
12^c^	**AAATCTTTGTTTGGT**GGCCGGCATGGTCCCAGCCTCC	F3 Vec F
13^c^	**CCAACACCCCGAGCG**CAACTCTCCAAGCGGCAATC	Vec-RFP-F
14^c^	**GCAGGACAGGGTTGT**AGCGAGAGAGGTAACGATTAG	Vec-RFP-R
15^d^	**AAACACGATAATACC**ATGTCTTCTGTATTCGATGAG	NP F
16^d^	**GTGGATCGGATCTTA**TCAGTACCCCCAGTCGGTGTC	NP R
17^d^	**AAACACGATAATACC**ATGGAGATGGCCACCTTTACAG	P F
18^d^	**GTGGATCGGATCTTA**TTAGCCATTCAGTGCAAGGCGC	P R
19^d^	**AAACACGATAATACC**ATGGCGAGCTCCGGTCCCGAG	L F
20^d^	**GTGGATCGGATCTTA**TTAAGAGTCACAGTTACTGTAG	L R
21^d^	**TAAGATCCGATCCAC**TAGTTCTAG	Vet up
22^d^	**GGTATTATCGTGTTT**TTCAAAGGAAAACC	Vet down
23^e^	TGGACCCTGGTTGTGCC	RFP dect F
24^e^	GGCTTCTTCATTCCCAACC	RFP dect R

All the primers were labeled with a superscript lowercase letter to better illustrate them. Nucleotides shown in bold letters represent homology sequences with a vector backbone, which were used to facilitate the RE independent cloning using the In-Fusion® PCR Cloning Kit (Clontech). ^a^Primers 1–6 were used to RT-PCR amplify the Fragment 1, Fragment 2, and Fragment3 genes from the viral RNA of NDV ZM10 strain. ^b^Primers 7–8 were used to amplify the RFP genes from LS-RFP plamid. ^c^Primers 9–14 were used to amplify or linearize the subclone vectors and the vector for RFP gene. ^d^Primers 15–22 were used to generate the helper plasmids of NDV strain ZM10. ^e^Primers 23–24 were used to amplify the insert part and a part of NDV vector gene

### Generation of helper plasmids of NDV strain ZM10

Viral genomic RNA of purified ZM10 virus was isolated with a MiniBEST Viral RNA/DNA Extraction Kit (TaKaRa Bio Inc, Japan). And three cDNA fragments NP, P, and L which contain the open reading frames (ORFs) of the nucleoprotein (N), phosphoprotein (P) and polymerase (L) were carried out by using a high-fidelity RT-PCR Reagent Kit (TaKaRa Bio Inc, Japan) with three pairs of specific primers separately. To clone the NP, P and L genes, three primer pairs of NP F and NP R, P F and P R, and L F and LR were used for PCR with PrimeSTAR® GXL DNA Polymerase (TaKaRa Bio Inc, Japan). These genes were then cloned into the protein expression plasmid pTM1, kindly provided by Dr. B. Moss [20]. The pTM1 vector was linearized by PCR with a pair of specific primers (Vet up and Vet down) with PrimeSTAR® GXL DNA Polymerase (TaKaRa Bio Inc, Japan). After gel purification, the fragments NP, P and L gene were cloned into the linearized pTM vector using the In-Fusion PCR cloning kit. Corresponding plasmids were designated as pZM10-NP, pZM10-P and pZM10-L, respectively.

### Construction of full-length clone of NDV harbouring RFP gene

To facilitate the RFP insertion into the NDV full-length clone as an additional transcription unit, a cDNA fragment containing the LaSota M noncoding sequences, gene end of M (GE) and gene start (GS) signals, Kozak sequence and the ORF of RFP protein were amplified by PCR from a recombinant plasmid LS-RFP (A gift from Dr Qingzhong Yu) by using the specific primer RFP-F and RFP-R, whereas primers Vec-RFP-F and Vec-RFP-R were used to amplify the linearized vector of pZM10. The RFP gene was inserted into the pZM10 between the P and M genes of the rZM10 genome to generate pZM10-RFP by using the In-Fusion PCR Cloning Kit, as shown in Fig. 1.

### Transfection and recovery of recombinant NDV

To generate recombinant viruses, BHK-21 cells were grown overnight in a six-well plate to approximately 80% confluency and infected with MVA/T7 at a multiplicity of infection (MOI) of 3 before the transfection. Subsequently, the cells were co-transfected with 2 µg of pTM-ZM10, 1 µg of pZM10-NP, 0.5 µg of pZM10-P, and 0.1 µg of pZM10-L by using 6µL Lipofectamine™ 3000 (Invitrogen) according to the manufacturer’s instructions. After incubation for 6 h at 37 °C, the transfection mixture was replaced and the cells washed once by PBS buffer and maintained in Opti-MEM medium containing 10% allantoic fluid (AF). After 72 h, the culture supernatant of each well was harvested, clarified and injected into the allantoic cavities of 9-day-old embryonated SPF eggs. 3 days later, AF was harvested and tested for NDV-specific haemagglutinating activity (HA). HA-positive viruses were filtered by 0.22 μM filter twice and diluted in phosphate-buffered saline (PBS), then inoculated into chicken embryos for 3 passages. Stocks were then prepared from harvested allantoic fluids. The complete genomic sequences of the rescued viruses were determined by direct sequencing of RT-PCR products amplified from the viral genomic RNA with the RT-PCR system we built before.

### Confirmation of the rescued viruses by western blot

DF-1 cells were infected with the recombinant NDV rZM10 and rZM10-RFP. After incubation for 48 h, the infected cells lysates were harvested. Cells were lysed and protein concentration was measured with BCA kit (Beyotime, China). 15 ug of total proteins obtained from the NDV infected DF-1 cells were subjected to SDS-PAGE. After trans-blotting, the membrane was incubated with mouse anti-HN protein monoclonal antibody (A gift from Dr Qingzhong Yu), rabbit anti-RFP tag ployclonal antibody (Genscript, USA), or mouse anti-β-actin monoclonal antibody followed by reactions with HRP-conjugatedgoat anti-mouse IgG secondary antibody and HRP-conjugatedgoat anti-Rabbit IgG secondary antibody (Beyotime, China). Then the expression of HN protein and RFP from each sample was measured with the Bradford assay kit (Bio-Rad, USA).

### Characterization of the rescued viruses

To evaluate the biological properties of the recombinant NDV rZM10 and rZM10-RFP, the pathogenicities and growth abilities of the recombinant viruses were examined by conducting titration assays and mean death time (MDT) and intracerebral pathogenicity index (ICPI) analysis. The virus titration was measured by the standard HA test in a 96-well microplate, the 50% tissue culture infectious dose (TCID_50_) assay on DF-1 cells, and the 50% egg infective dose (EID_50_) assay in 9-day-old SPF chicken embryos, and ICPI was determined in 1-day-old chickens, according to standard procedures [21].

To analyze the growth kinetics of the parental virus and the recombinant viruses, monolayer of DF-1 cells (in triplicate) was infected with the each virus at 0.01 MOI, respectively. The virus lysates was harvested at 12h intervals, and the virus titer of each time point was measured by TCID_50_ titration on DF-1 cells in triplicate from two independent experiments.

To determine the genetic stability of the additional gene in the recombinant NDV genome, ten successive passages of rZM10-RFP in 9 days old SPF embryonated chicken eggs were performed. Briefly, the egg-passaged 3 (EP3) rZM10-RFP virus was diluted 10^5^ fold with phosphate buffer solution (PBS) and 100 µL of the diluted EP3 inoculated into the allantoic cavity of each embryo. Three independent repetitive groups (A, B, and C) were set for each passage with 3 eggs for each group. The AF of the infected eggs was harvested and pooled within each repetition group at 96 h post-infection. The egg-passaged viruses were diluted 10^5^ fold, and passaged on their corresponding groups of eggs for six more times. The serially egg-passaged viruses were designated as EP4 (A, B or C) to EP10 (A, B or C), respectively. Then the genomic RNA was extracted from the allantoic fluid of EP4, EP6, EP8, and EP10. The parental rZM10 virus stock were used to amplify the RFP insert by RT-PCR with the specific primer RFP dect F for the RT stage using the PrimeScript™ II 1st Strand cDNA Synthesis Kit (TaKaRa Bio Inc, Japan) and a pair of specific primers (RFP dect F and RFP dect R) for the PCR program with PrimeSTAR® GXL DNA Polymerase (TaKaRa Bio Inc, Japan). The RT-PCR products were analyzed by electrophoresis on 1% agarose gel and photographed using a Tanon-1600 Imager (Tanon, China).

## Results

### Construction of full-length cDNA clone of NDV strain ZM10

In this study, the genome of NDV ZM10 strain was successfully cloned into the pBluescript and the RFP gene was inserted into pZM10 between the P and M genes as an independent transcription unit as shown in Fig. 1. To generate the vector pZM10-RFP, the RFP cassette was introduced in the non-coding region of the vector pZM10 between the P and M using In-Fusion® PCR cloning techniques. The total size of the pZM10-RFP was found to be 16 092 n, followed ‘the rule of six’.

### Rescue of recombinant NDV from cloned cDNA

**Fig. 2:**
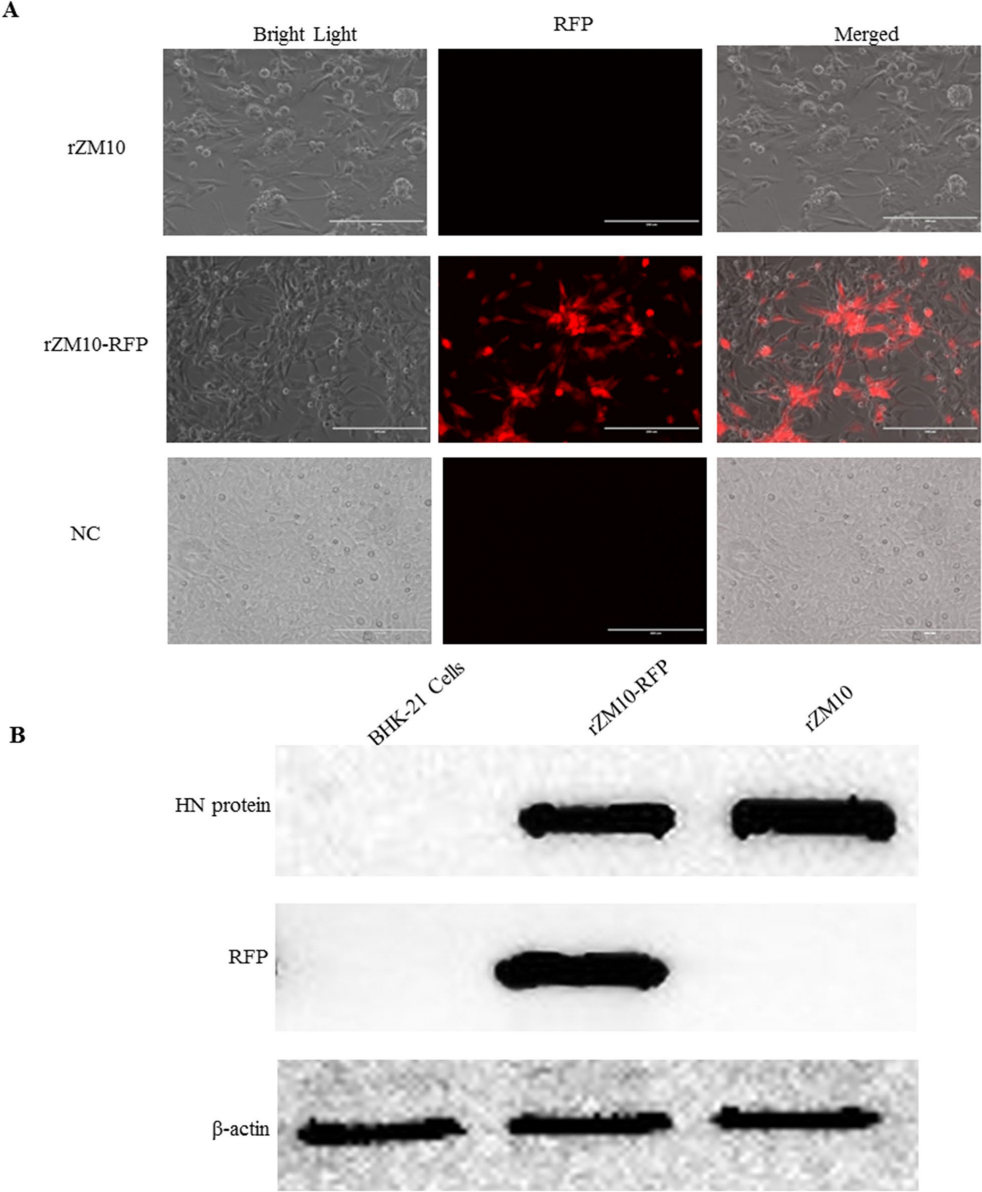
The CPEs and expression of RFP by the recombinant viruses rZM10 and rZM10-RFP. **A** DF-1 cells in a 6-well plate were infected with the recombinant viruses at 0.01 m.o.i. the CPEs and the fluorescence of the infected cells were examined and digitally photographed 48 h post-infection, under an inverted fluorescence microscope at 100 × magnification. The DF-1 cells which were not infected was set up as negative control. **B** Western blotting analysis antibodies response in DF-1 cells infected with the recombinant viruses rZM10 and rZM10-RFP. The molecular weight of HN protein is approximately 87 kDa and was present in the cells infected with the rZM10 or rZM10-RFP. The RFP(~ 30 kDa) protein bands could only be detected in the rZM10-RFP infected cells, confirming the expression of the RFP gene by rZM10-RFP

Rescue of the recombinant rZM10 or rZM10-RFP virus was performed by co-transfecting the plasmids pZM10 or pZM10-RFP respectively with the supporting plasmids pZM10-NP, pZM10-P and pZM10-L together into BHK-21 cells. The rescued viruses were amplified by inoculating 200 µL of the infected cell lysate into the 9-day-old SPF chicken embryos’s allantoic cavity and incubating for 96 h at 37 °C. Then the rescued viruses were collected by harvesting the AF and the virus titer was further detected by hemagglutination (HA) assay. The rescued virus was diluted and propagated for three times in the SPF chicken embryos. The AF was harvested after the propagation and stored at − 80 °C. The insert gene and the viral genome of the rescued virus were determined by sequencing the RT-PCR products amplified from the AF. Besides, the viral cytopathic effect (CPE) produced in the BHK-21 cells typically consisted of clumping and rounding of cells and fusion of cells with syncytia formation as observed 48 hpi under the light microscope(Fig. 2A). The expression of RFP could be observed as a bright red fluorescence under a fluorescent microscope at 200 × magnifications (Nikon, Eclipse Ti, Melville, NY) (Fig. 2A).

### Confirmation of the recombinant viruses

In order to confirm the viruses, rescued viral RNA was extracted from fresh allantoic fluid, and the full-length genome was sequenced. It was found that the genomic sequence of the recombinant virus was identical to that of the parental virus, with only two exceptions: G at nt 8405 instead of the original A and A at nt 11209 instead of G. Both mutations were located in the open reading frame of the L protein sequence. The codon at nt 8405 was changed from A (Ala) to T (Thr) while the mutation at nt 11209 caused no amino acid change. As the rZM10 or rZM10-RFP have kept the similar biological characterization compared to their parental virus (see the “Results” section), it seem the mutation have no influence on the recombinant virus, which could be considered a genetic marker of rescued virus particles.

The expression of NDV HN protein in the recombinant NDV infected DF-1 cells were analyzed. As shown in Fig. 2B, western blot analysis showed that the HN protein had a molecular weight of approximately 67 kDa and were present in the cells infected with the rZM10 or rZM10-RFP, indicating that the recombinant NDV was successfully rescued. While the RFP(~ 26 kDa) protein bands could only be detected in the rZM10-RFP infected cells, confirming the expression of the RFP gene.

### Biological and molecular characterization of the recombinant viruses

To evaluate biological properties of the parental and recombinant viruses on viral pathogenicity and growth dynamics, the recombinant NDV viruses rZM10 and rZM10-RFP were examined in vitro and in vivo by testing the virus titration (EID_50_, TCID_50_ and HA test), mean death time (MDT), and intracerebral pathogenicity index (ICPI). As shown in Table 2, the rescued viruses rZM10 and rZM10-RFP showed similar attenuated characteristics in SPF embryonated eggs and day-old chickens with a long MDT (> 150 h) and low ICPI (0.0) compared to the parental strain ZM10. The titers of the recombinant viruses grown in embryonated eggs were measured by EID_50_, TCID_50_ and HA. The results showed that the recombinant virus rZM10-RFP were slightly lower but were comparable to that of rZM10 and ZM10 strain.

**Table 2:** Biological assessments of the NDV recombinant viruses

Viruses	MDT	ICPI	HA	EID_50_	TCID_50_
ZM10	> 150hs	0.0	2^10^	3.16 × 10^9^	1.78 × 10^8^
rZM10	> 150hs	0.0	2^10^	3.16 × 10^9^	1.78 × 10^8^
rZM10-RFP	> 150hs	0.0	2^9^	5.62 × 10^8^	4.33 × 10^7^

**Fig. 3:**
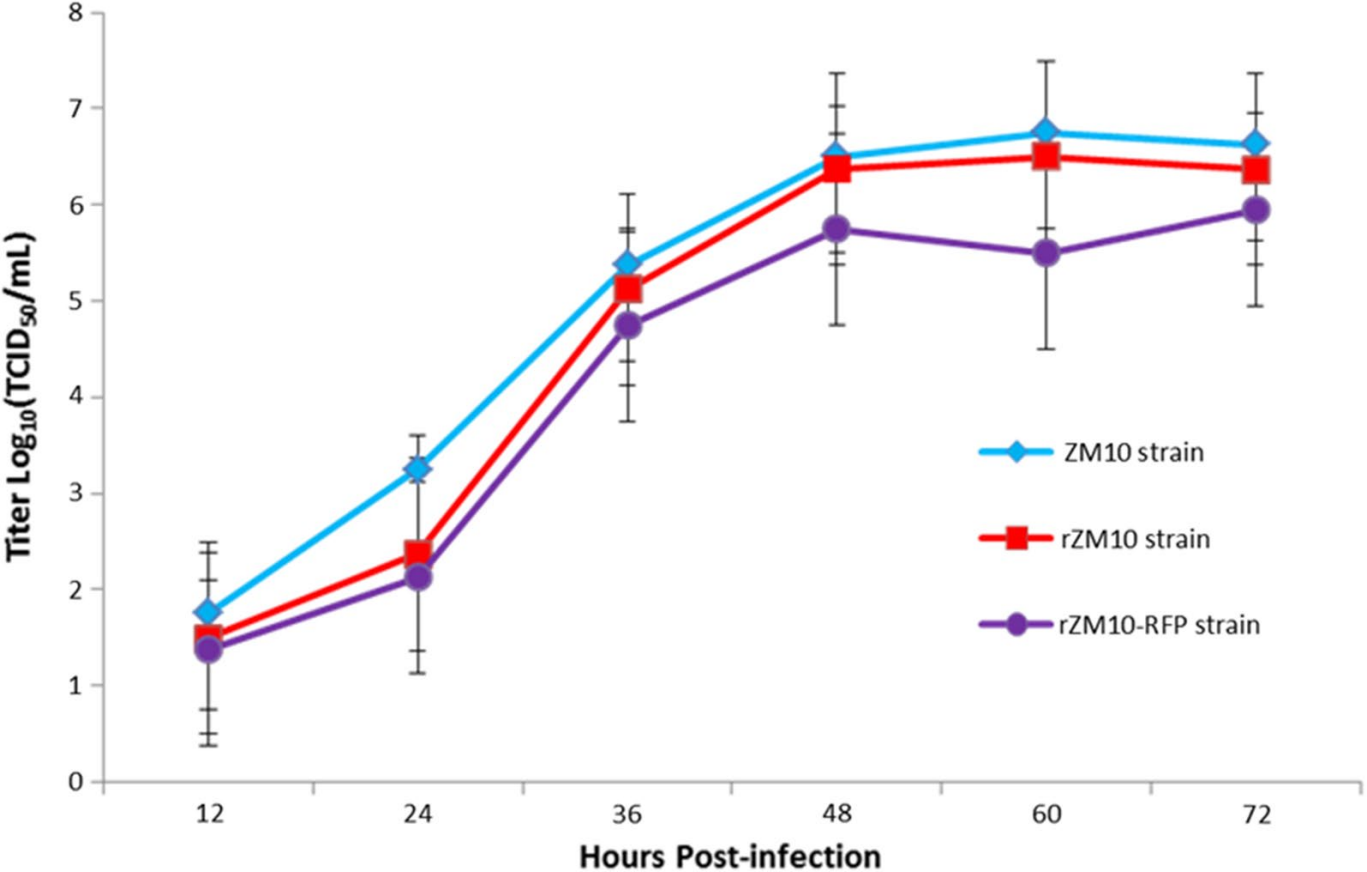
Growth kinetics of the recombinant viruses rZM10 and rZM10-RFP. DF-1 cells were infected with the indicated NDV viruses at 0.01 MOI. Every 12 h post-infection, virus lysates were harvested. Virus titers were measured by TCID_50_ titration on DF-1 cells for each time point in triplicates from two independent experiments and expressed in mean log10 TCID_50_/mL with a deviation

As shown in Fig. 3, the growth kinetics study was carried out on DF-1 cells with a collection interval of 12 h until 72 h of infection. The growth kinetics of the two recombinant viruses was slightly delayed in the onset of replication compared to the parental virus ZM10, however, after 36 h, the recombinant viruses displayed a comparative replication kinetics and magnitude. The rescued virus rZM10 and rZM10-RFP had a similar kinetic and replicative efficiency as evident by the growth curve, indicating that the insertion of the RFP gene did not influence the viral replication of NDV (Fig. 3).

**Fig. 4:**
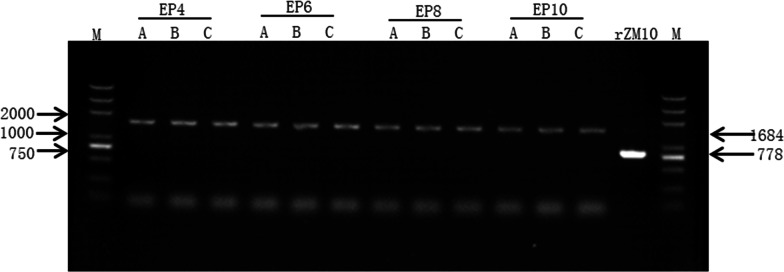
Detection of the RFP gene in egg-passaged virus stocks by RT-PCR and gel electrophoresis. Viral RNAs were extracted from the EP4, 6, 8 and 10 stocks (A, B, and C), and rZM10 as control. The RFP gene insert and surrounding sequences in the NDV vector were amplified from viral RNAs by using a PrimeScript™ II 1st Strand cDNA Synthesis Kit (TaKaRa Bio Inc, Japan) and a pair of specific primers. The RT-PCR products were analyzed by electrophoresis on 1% agarose gel and photographed using a Tanon-1600 Imager (Tanon, China). The sizes of the DL2 000 Plus DNA Ladder marker (Zoman Bio, China) are labelled with black arrows on the left side of the gel. The predicated sizes of the RT-PCR products from the rZM10-RFP (1685 bp) and the rZM10 control (778 bp) are labeled with black arrows on the right side of the gel

To determine the genetic stability of the rZM10-RFP virus, RT-PCR was carried out to test the inserted gene RFP of rZM10-RFP EP4, EP6, EP8, and EP10. The predicted molecular weight is 1684 bp which includes the RFP cassette (906 bp) and a part of the recombinant ZM10 vector (778 bp). As shown in Fig. 4, the RT-PCR products amplified from the EP4, EP6, EP8, and EP10 of rZM10-RFP virus (A, B, and C) migrated to approximately 1.7 Kb on the agarose gel, which corresponds to the predicted molecular weight. While the RT-PCR product amplified from the rZM10 virus migrated to the proximity of the predicted molecular weight position (778 bp). These result indicated that there was no gross noticeable deletion/insertion in the rZM10-RFP virus insertion region.

## Discussion

In this study, the reverse genetics platform for enterotropic Newcastle disease virus strain ZM10 and rZM10, expressing an inserted foreign gene RFP was established. The reverse genetics system for NDV is difficult and complex compared to DNA viruses or positive-sense RNA viruses [22–24]. First of all, the naked genomic RNA of NDV is not infectious. It is only functional when being encapsidated with protein complex, ribonucleoprotein (RNP) formed by the viral NP, P and L proteins. Secondly, the genome length and genome complexity have made that the cloning of the full-length NDV genome remains the most challenging and laborious of all the steps [23, 24]. Moreover, the NDV genome has to meet particular requirements for successful rescue such as the rule-of-six [25, 26] and generation of the precise 3′ and 5′ ends [24, 26]. To get the precise integral NDV genome, the traditional method is dividing the NDV genome into a set of short fragments from 6 to 11 [10, 14, 27–31], and it takes months to complete for the multiple DNA fragments cloning [23]. In this study, the genome of NDV was divided into three fragments 6, 6 and 3 kb. Compared to the approaches before, the design in this study needs much shorter time for the full-length genome cloning. As the amplification of a cDNA fragment around 10 kb is available by using the commercial high fidelity reverse transcription PCR by now, it seems the strategy of dividing the whole NDV genome into two parts is possible, which need to be explored in the future.

A majority of recombinant NDVs expressing one foreign gene (FG) as vaccines or biological therapies express through either an independent transcription unit (ITU) or an internal ribosomal entry site (IRES) approach [29, 32, 33]. The efficacy of these two strategies has been investigated, showing that the ITU approach was more efficient than that through the IRES approach [32]. The expression levels of FGs through ITU at different genomic locations were variable and the optimal insertion site in NDV genome for efficient expression has been identified as the non-coding region between the P and M genes [16, 17, 34]. Here, the RFP gene was inserted into the NDV genome at the site of the non-recoding region between P and M gene. Our data showed that the RFP could be stably expressed in a high level in this region (Fig. 4), and the recombinant virus rZM10-RFP did not showed any significantly differences in MDT and ICPI (Table 2), indicating that the noncoding region between the P and M genes is a suitable position for rZM10 to express an FG and provides the design strategy for the recombinant bivalent vaccines.

Pathogens especially the viruses could infect corresponding host cell types, results in replicating in specific host organs [35–37]. The ZM10 strain has an obvious preference for replication the intestine though it could infect both the respiratory and intestinal tract. This characteristic makes the ZM10 to be an ideal vector to express the protective antigen of intestinal pathogens, therefore, an important application of our NDV rescue platform would be to use the recombinant viruses as vaccine vector for the expression of protective antigens against avian or other animals’ diseases to develop multivalent vaccines (Additional file 1).

## Conclusions

In this study, the full-length genome of NDV ZM10 strain was cloned and the reverse genetic system for ZM10 was established. The recombinant ZM10 could express the RFP and both the rZM10 and rZM10-RFP showed similar biological properties and growth kinetics compared to the parental virus. Considering that the ZM10 is an avirulent enterotropic NDV strain, our research provides an ideal vector for developing recombinant multivalent vaccines, especially for the pathogens grows in the intestine.

## Supplementary Information

**Additional file 1.** **Figure S2-B1.** Western blotting result of NDV HN protein expression. **Figure S2-B2.** Western blotting result of RFP protein expression. **Figure S2-B3.** Western blotting result of β-actin protein expression. **Figure S4.** Detection of the RFP protein in egg-passaged (EP) virus stocks (EP4, 6, 8 and 10) by RT-PCR and gel electrophoresis.

## Data Availability

All authors declare that the data supporting the funding of this study are available within this article. The sequences information of the recombinant ZM10-RFP and the helper plasmids pTM-N/P/L are available in GenBank. For plasmid pZM10-RFP, its accession number is OP215547 and the link is https://www.ncbi.nlm.nih.gov/nuccore/OP215547.1; The accession numbers and the links of the three helper plasmids pTM-N/P/L are OP215548(https://www.ncbi.nlm.nih.gov/nuccore/OP215548), OP215549(https://www.ncbi.nlm.nih.gov/nuccore/OP215549), and OP215550(https://www.ncbi.nlm.nih.gov/nuccore/OP215550), respectively.

## Abbreviations

ND: Newcastle disease
RFP: Red fluorescent protein
LIC: Ligation-independent cloning
ORFs: Open reading frames
MOI: Multiplicity of infection
AF: Allantoic fluid
CPE: Cytopathic effect
FG: Foreign gene
ITU: Independent transcription unit
IRES: Internal ribosomal entry site
