# Supplementary material for: Rescue of an enterotropic Newcastle disease virus strain ZM10 from cloned cDNA and stable expressing an inserted foreign gene
Source: BMC Biotechnol. 2022 Dec 6;22:38. doi: 10.1186/s12896-022-00763-5 (PMC9724440; doi:10.1186/s12896-022-00763-5)

## Supplementary Information

Original image description of all the gels or immunoblots involved in the article.

Figure S2-B1. Western blotting result of NDV HN protein expression

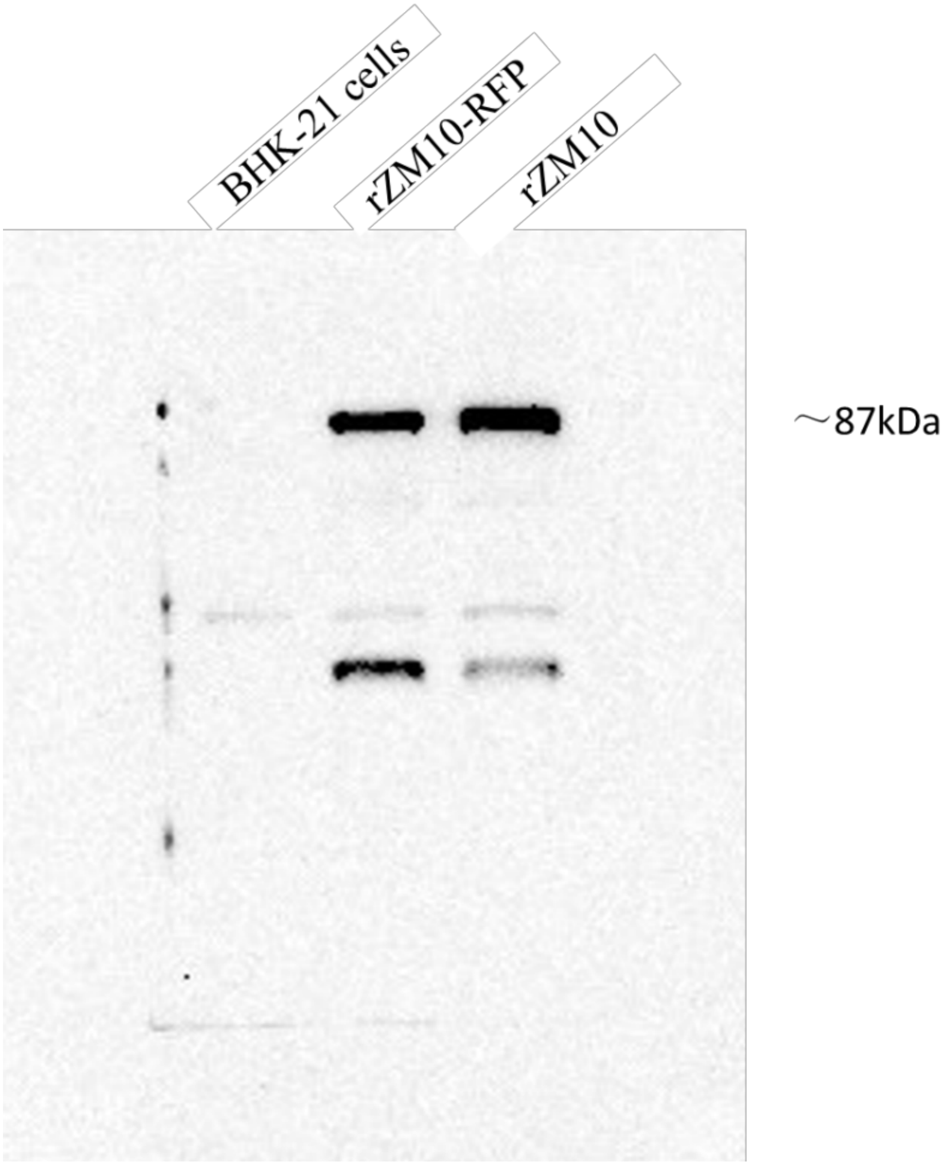


Figure S2-B2. Western blotting result of RFP protein expression

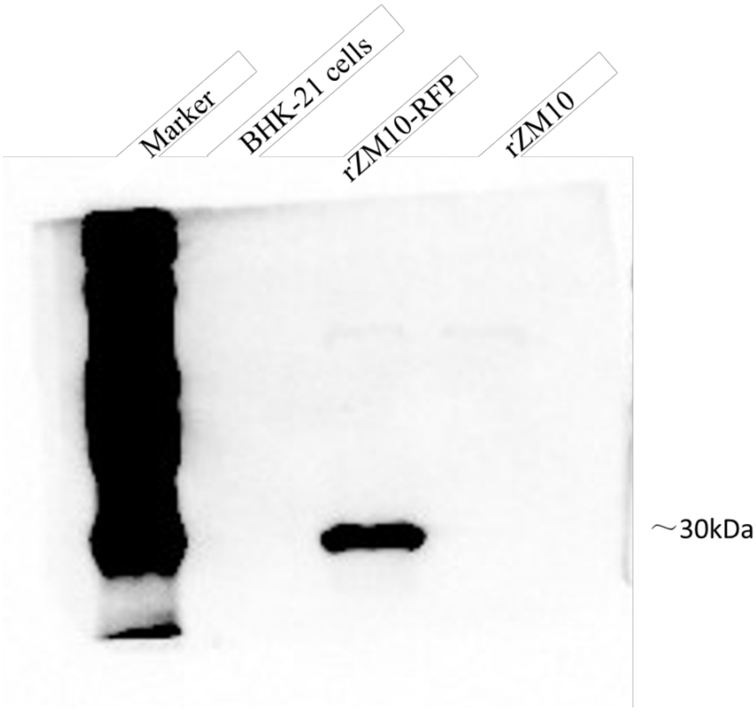


Figure S2-B3. Western blotting result of β-actin protein expression

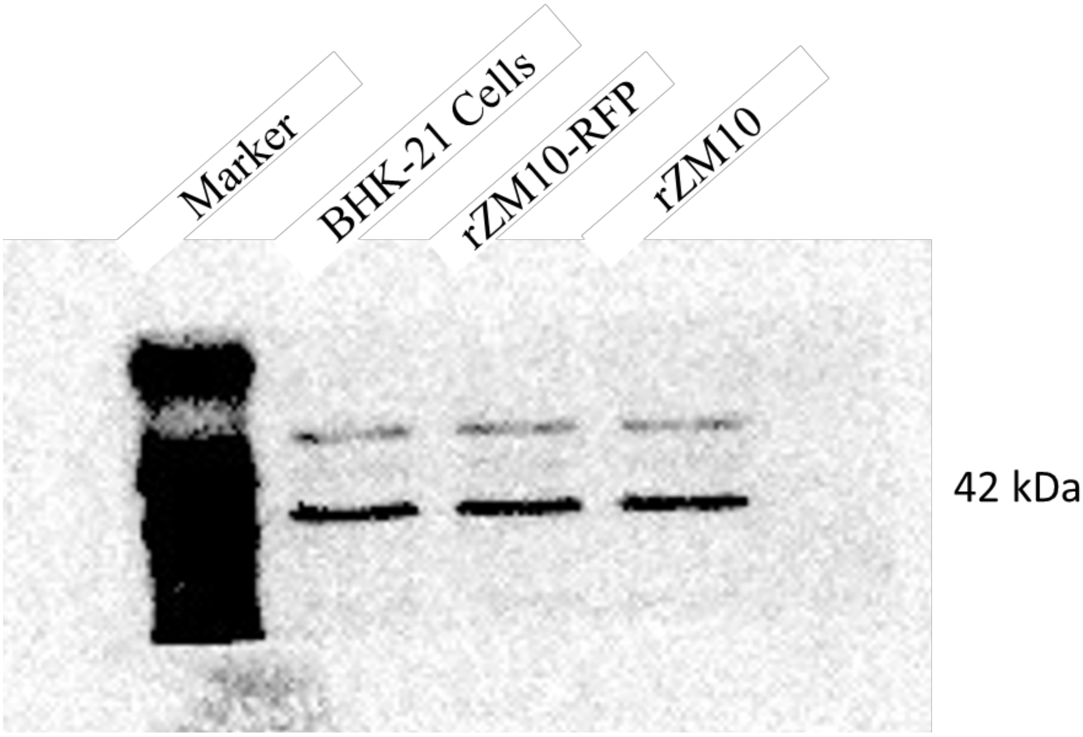

Figure S4. Detection of the RFP protein in egg-passaged (EP) virus stocks (EP4, 6, 8 and 10) by RT-PCR and gel electrophoresis.

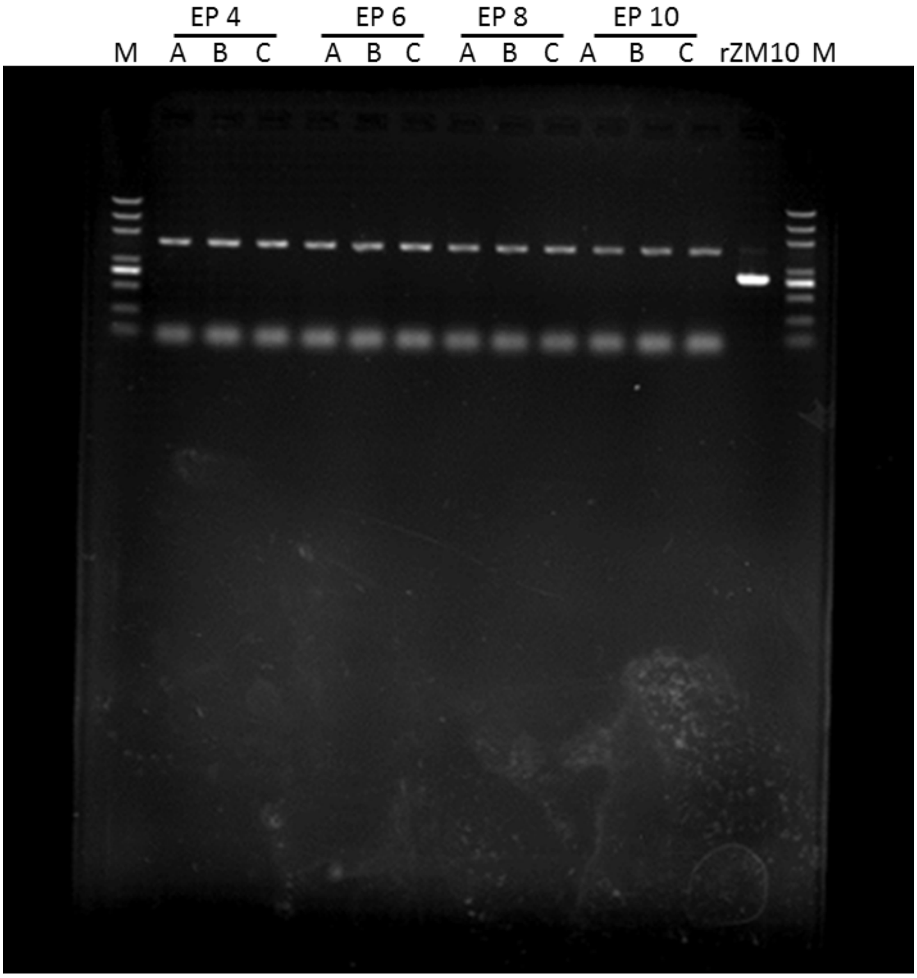

Supplement: Supplementary file 1 — Additional file 1. Figure S2-B1. Western blotting result of NDV HN protein expression. Figure S2-B2. Western blotting result of RFP protein expression. Figure S2-B3. Western blotting result of β-actin protein expression. Figure S4. Detection of the RFP protein in egg-passaged (EP) virus stocks (EP4, 6, 8 and 10) by RT-PCR and gel electrophoresis. [file 12896_2022_763_MOESM1_ESM.pdf]
